# Editorial: Diagnostic and treatment strategies for periodontal disease

**DOI:** 10.3389/fdmed.2025.1759435

**Published:** 2026-01-12

**Authors:** C. Janakiram, R. M. Baiju, L. Puzhankara, H. R. Rajeshwari

**Affiliations:** 1 Department of Public Health Dentistry, Amrita School of Dentistry, Amrita Vishwavidyapeetham, Kochi, India; 2 Department of Periodontology, Government Dental College, Kerala University of Health Sciences, Kottayam, India; 3 Department of Periodontology, Manipal College of Dental Sciences, Manipal Academy of Higher Education, Manipal, India; 4 Royal College of Dental Surgeons of Ontario, Toronto, ON, Canada

**Keywords:** diagnostic methods, periodontal therapy, periodontal pathogenic bacteria, periodontitis, treatment strategy

## Introduction

“It amounts to a truism to say that progress in the practical arts of medicine in any of its branches, whether preventive or curative, only comes from the growth of accurate knowledge as it accumulates in the laboratories and studies of the various sciences.”

— Walter Fletcher

Periodontal health forms a vital component of overall oral and general health. Precise diagnosis, accurate classification, and timely intervention are fundamental to establishing and maintaining a healthy periodontium. Staying updated with current evidence on advancements in diagnostic and therapeutic modalities is crucial for achieving optimal periodontal health.

## Original articles (5) and brief research report (1)

Periodontal disease diagnosis and treatment have been evolving over time. Conventional diagnostic methods are being gradually replaced with advanced diagnostic methods. The use of biomarkers as a diagnostic tool can facilitate early identification of progression of destructive periodontal disease. In their study, Padalkar et al., have shown that salivary Periostin levels are associated with periodontal disease and can act as a non-invasive biomarker for periodontal disease. The paper reports that salivary periostin may be utilized for evaluating both healthy and diseased periodontium.

Data obtained from biomarker assessment; clinical assessments must be collated and interpreted to achieve reliable results. Accurate interpretation of the data is essential to arrive at an unambiguous diagnosis and plan the most appropriate treatment. Artificial Intelligence (AI) is a rapidly changing gargantuan entity encompassing nearly all aspects of life and capable of efficient assimilation of data and arriving at near precise conclusions. In the brief research report by Nantakeeratipat et al., Google Cloud's Vertex artificial intelligence (AI) automated machine learning (AutoML) has been used to develop a model for detecting dental plaque levels on permanent teeth. Vertex AI AutoML was used for non-invasive detection of dental plaque and the two-class acceptable-unacceptable model using the AI demonstrated improved performance in revealing the presence of dental plaque. Alveolar bone loss assessment and predicting individualized periodontal prognoses using AI has been studied by Jundaeng et al. The AI model was shown to be a faster, more accurate, and requires less rigorous manual effort to conventional methods of determining diagnosis and prognosis. These two articles on AI demonstrates the significance of using AI in determining the diagnosis of periodontal disease and in estimating the prognosis.

Management of periodontal disease involves the use of mechanical and chemical methods. The use of phytonutrients in periodontal therapy has been shown to be equivalently effective in managing periodontal diseases as other antimicrobials. The study by Bartels et al. tested Mentha aquatica, Mentha longifolia, Sideritis euboea, Sideritis syriaca, Stachys spinosa, Satureja parnassica, Satureja thymbra, Lavandula stoechas, Achillea taygetea, Phlomis cretica, and Vaccinium myrtillus against oral pathogens *Streptococcus mutans, Streptococcus oralis, Streptococcus sobrinus, Prevotella intermedia, Fusobacterium nucleatum, Parvimonas micra, Porphyromonas gingivalis, and Candida albicans*. All extracts, except the methanol extract of *V. myrtillus*, showed an antibacterial effects at concentrations ranging from 10 to 0.15 mg/ml while the extracts did not have antifungal effects. The anaerobic pathogens were more susceptible to bacteriostatic and bactericidal activity at lower concentrations compared to the aerobic pathogens. Biofilm formation by *S. mutans* was inhibited by extracts of *L. stoechas*, *S. thymbra*, *S. parnassica*, and the methanol extract of *V. myrtillus* were effective at concentrations up to 1.25 mg/ml. Although the biofilm inhibition by *P. cretica* decreased at 2.5 mg/ml, it was able to inhibit and kill *S. mutans* at a concentration of 0.6 mg/ml. The findings from the study emphasized the importance of phytonutrients as antimicrobial agents against oral pathogens.

Mechanical therapy has been the cornerstone for periodontal disease management. Open flap debridement, used for treating patients with advanced periodontal disease, may have complications. Identifying key risk factors for these complications can help develop more refined surgical management strategies. The retrospective study by Gu et al. recognized age, clinical attachment loss, smoking, and surgical incision type as significant predictors of complications following periodontal open flap debridement surgery. The findings highlight the need for customized surgical approaches which takes into consideration the risk factors for surgical complications such as age, severe periodontal disease and habits like smoking.

## Review articles (2)

Research is an essential component to determine the efficacy of any diagnostic method or therapy. Prior to human clinical studies, often *in vitro* studies are performed to ensure safety and effectiveness. Various species have been employed in periodontal research due to their biological similarities to humans. These models facilitate research on disease progression, and potential therapeutic strategies in a controlled environment. The review by Barik et al. evaluates the strengths and limitations of animal models used in periodontal disease studies. Although non-human primates have the greatest similarities with humans, they are expensive and challenging to care for and not practical to utilize for both fundamental scientific research and treatment investigations on periodontal disease. Rodents are more cost-effective and simpler to manage. However, they do not replicate the course of periodontitis completely in humans. They may be useful for understanding some aspects of the host-microbial interaction, and therapy. While rodents may not replicate human periodontal disease perfectly, they can be valuable aids in providing insights into the disease process and potential treatments. The review emphasizes the need for refinement of animal models to enhance their importance to human conditions.

One of the prevalent causes of oral discomfort is food impaction which has a multifactorial etiology and can lead to a cascade of complications like periodontal disorders and caries. The review by Ma et al. explores the classification and treatment of food impaction, focusing on vertical and horizontal types. Identifying the underlying cause is essential for effective management of food impaction. Restoring adjacent contacts, adjusting occlusion, reshaping teeth or prostheses, and modifying both adjacent and opposing teeth can help in vertical impaction of food. Horizontal food impaction requires periodontal management including interdental cleaning and maintenance, reconstruction of periodontal osseous defects, papillary reconstruction, restorative and prosthetic management of dentition deficiencies.

Together, this set of articles offers the reader a diverse perspective on the various diagnostic and treatment strategies for periodontal disease and pave the way for advances in the field.

